# Molecular and functional interactions of alpha-synuclein with Rab3a

**DOI:** 10.1016/j.jbc.2022.102239

**Published:** 2022-07-06

**Authors:** Guohua Lv, Myung Soo Ko, Tapojyoti Das, David Eliezer

**Affiliations:** Department of Biochemistry, Weill Cornell Medical College, New York, New York, USA

**Keywords:** alpha-synuclein, Rab3a, NMR, membrane protein, Parkinson’s disease, a-Syn, alpha-synuclein, GAP, GTP-hydrolysis activating protein, HSQC, heteronuclear single quantum coherence, MTSL, 1-oxyl-2,2,5,5,-tetramethylpyrroline-3-methyl-methanethiosulfonate, PD, Parkinson’s disease, PTM, posttranslational modification, PRE, paramagnetic relaxation enhancement, SUV, small unilamellar vesicle, SV, synaptic vesicle

## Abstract

Alpha-synuclein (a-Syn) is a presynaptic protein, the misfolding of which is associated with Parkinson’s disease. Rab GTPases are small guanine nucleotide binding proteins that play key roles in vesicle trafficking and have been associated with a-Syn function and dysfunction. a-Syn is enriched on synaptic vesicles, where it has been reported to interact with GTP-bound Rab3a, a master regulator of synaptic vesicle trafficking. a-Syn is known to bind weakly to Rab8a in solution *via* a positively charged patch, but the physiological implications of such interactions have not been explored. Here, we investigate direct interactions between a-Syn and Rab3a in solution and on lipid membranes using NMR spectroscopy. We find that the C terminus of a-Syn interacts with Rab3a in a manner similar to its previously reported interaction with Rab8a. While weak in solution, we demonstrate that this interaction becomes stronger when the proteins are bound to a membrane surface. The Rab3a binding site for a-Syn is similar to the surface that contacts the Rab3a effector rabphilin-3A, which modulates the enzymatic activity of Rab3a. Accordingly, we show that a-Syn inhibits GTP hydrolysis by Rab3a and that inhibition is more potent on the membrane surface, suggesting that their interaction may be functionally relevant. Finally, we show that phosphorylation of a-Syn residue Ser 129, a modification associated with Parkinson’s disease pathology, enhances its interactions with Rab3a and increases its ability to inhibit Rab3a GTP hydrolysis. These results represent the first observation of a functional role for synuclein-Rab interactions and for a-Syn Ser 129 phosphorylation.

Parkinson's disease (PD) is the second most common neurodegenerative disorder after Alzheimer’s disease. The protein alpha-synuclein (a-Syn) is the major component of intraneuronal proteinaceous aggregates known as Lewy bodies (1) that are a diagnostic hallmark of PD. A-Syn belongs to the class of intrinsically disordered proteins and undergoes a disorder-to-helix transition upon binding to membranes and membrane mimetics (2, 3). The physiological functions of a-Syn remain to be conclusively established, but evidence suggests that a-Syn is involved in the regulation of synaptic vesicle (SV) exocytosis (4, 5, 6, 7, 8, 9, 10, 11, 12) and that direct interactions with lipids and SVs are important for a-Syn function. In addition, phosphorylation at residue Ser 129, located in the acidic C-terminal tail of the protein, is the most abundant posttranslational modification (PTM) of a-Syn in PD (13, 14). In Lewy bodies, 90% of a-Syn is estimated to be phosphorylated at Ser 129 (14). However, the functional role and consequences of phosphorylation at this site are largely unknown (15, 16).

Rab proteins are a subfamily of the small GTPases superfamily, which function as molecular switches that interact with downstream effector proteins in their activated, GTP-bound state and initiate a wide spectrum of cellular signaling pathways (17). Hydrolysis of GTP to GDP leads to dissociation of the effector proteins and terminates downstream events (17, 18, 19, 20, 21). Regulation of Rab GTPase activity provides a mechanism for controlling Rab signaling and is affected by GDP-GTP exchange factors and GTP-hydrolysis activating proteins (GAPs). GDP-GTP exchange factors and GAPs typically influence Rab activity by binding to the functionally critical switch-I and switch-II regions, which undergo conformational transitions during catalysis that are critical for GTP hydrolysis. Rab proteins play a key role in intracellular vesicular trafficking events (13, 22, 23) and have been associated with a-Syn–related neuronal function and dysfunction (8, 24, 25, 26, 27, 28, 29, 30, 31, 32). For example, overexpression of Rab8a, Rab1, and Rab3a attenuate a-Syn-induced cytotoxicity in cellular and animal models of PD, suggesting a functional interplay between Rab GTPases and known PD factors (8, 33). In addition, a-Syn cycling on and off SVs was reported to be influenced by the Rab3a machinery. Specifically, membrane-associated GTP-bound Rab3a stabilizes a-Syn on SVs, and the GDP dissociation inhibitor-Hsp90 complex that controls Rab3a membrane dissociation also regulates a-Syn dissociation from membranes (34).

Here, we investigate in detail the interaction of a-Syn with GTP-bound Rab3a in solution and on the surface of small unilamellar vesicles (SUVs) using NMR spectroscopy. We show that the C terminus of a-Syn binds to a positively charged patch on the surface of GTP-bound Rab3a that includes the functionally important switch-I and -II regions and that closely resembles the Rab3a binding epitope responsible for its interactions with one of its functional effectors, rabphilin-3A. This interaction is weak in solution but stronger when both proteins are localized to the membrane surface, as would be the case *in vivo*. Measurements of Rab3a enzymatic activity in the absence and presence of a-Syn reveal that a-Syn is an inhibitor of Rab3a GTP hydrolysis, delineating, for the first time, a potential functional role for this interaction *in vivo*. Phosphorylation of a-Syn at residue Ser 129 (pS129 a-Syn) increases its affinity for Rab3a and enhances its inhibition of Rab3a activity, suggesting a potential functional role of this disease-associated PTM.

## Results

### The C terminus of a-syn interacts with GTP-bound Rab3a

To characterize whether and how a-Syn binds directly to Rab3a, we used NMR spectroscopy, a versatile tool for the analysis of protein–protein or protein–ligand interactions (35). We used an N-terminally 6-His-tagged construct (a kind gift from Dr David Lambright) encompassing the GTPase domain of Rab3a (residues 15–186), because the N and C termini of the protein are important for prenylation and targeting to membranes but are not considered necessary for nucleotide binding and hydrolysis or for interactions with effectors or regulatory proteins (36, 37, 38). We introduced the Q81L mutation into our Rab3a construct because GTP hydrolysis is impaired by this mutation (39) and Rab3a was reported to interact with a-Syn preferentially in its GTP-bound state (34). Finally, to prevent concerns pertaining to cysteine oxidation or cross-linking, we also mutated the only Cys residue in Rab3a to Ala (C137A mutation). All experiments reported, except for the GTPase activity assays, were performed using this construct, which is henceforth simply referred to as Rab3a.

**Figure 1:**
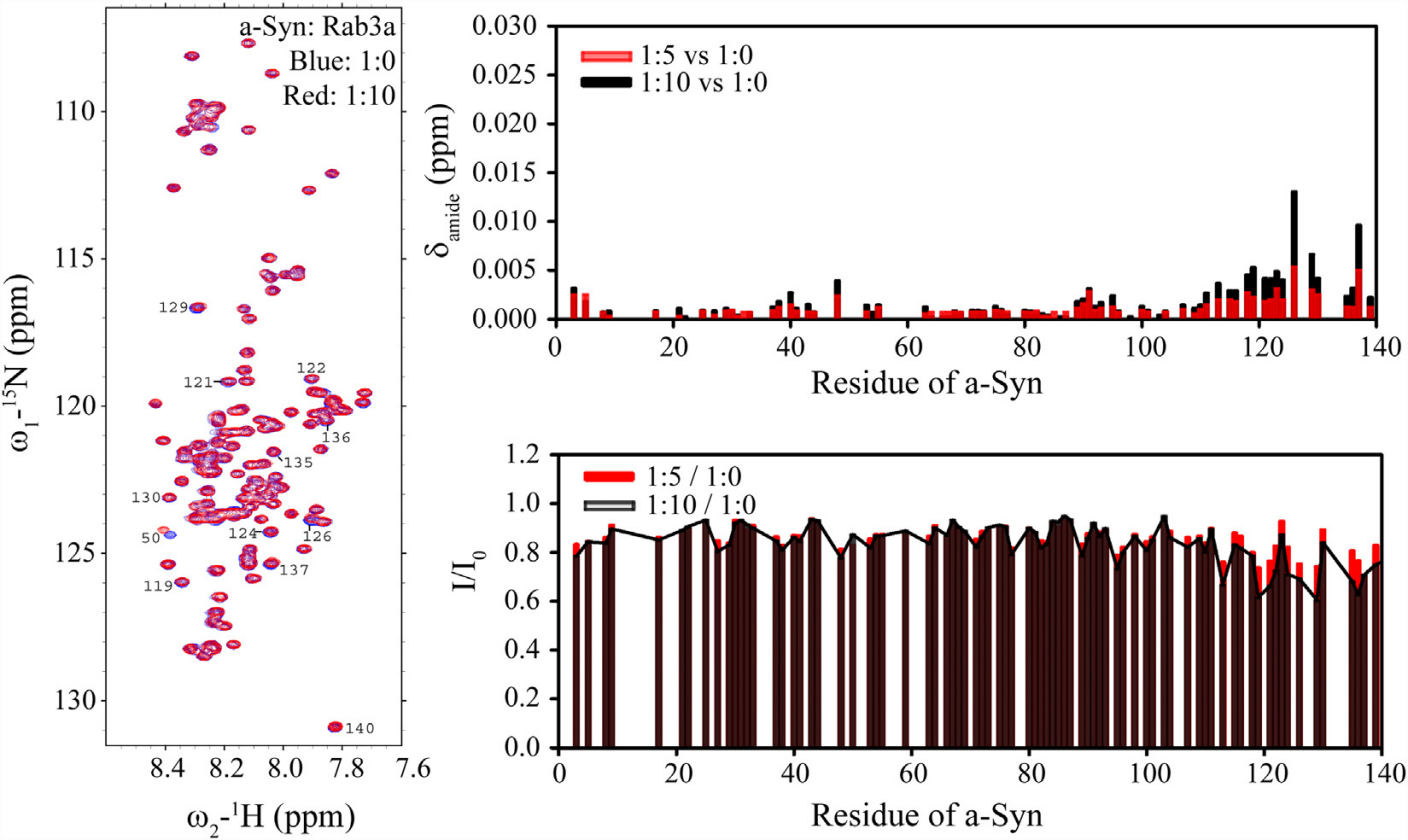
**GTP-bound Rab3a binds to the C terminus of**^**15**^**N-labeled a-Syn.** NMR ^1^H-^15^N HSQC spectra of ^15^N-labeled a-Syn in the absence (*left panel*, *blue*) or presence of 10-fold excess GTP-bound Rab3a (*left panel*, *red*) exhibit only small changes. Chemical shift changes of the amide cross peak (Δδ_amide_ = √1/2(Δδ_ΗΝ_^2^ + (Δδ_Ν_/5)^2^)) upon addition of 5-fold (*red*) or 10-fold (*black*) excess Rab3a are observed at the C-terminus of the protein (*right upper panel*). Decreased NMR signal intensity ratios are also observed at the C terminus in the presence of 5-fold (*red*) or 10-fold (*black*) excess Rab3a (*right lower panel*). a-Syn, alpha-synuclein; HSQC, heteronuclear single quantum coherence.

We monitored two-dimensional ^1^H–^15^N correlation spectra (heteronuclear single quantum coherence, HSQC) of ^15^N-labeled a-Syn in the presence of increasing amounts of unlabeled GTP-bound Rab3a for changes in chemical shifts and signal intensities. Slight changes in the NMR signals for C-terminal a-Syn residues ranging from L113 to A140, including small chemical shift changes and small intensity decreases (Fig. 1, left panel), became detectable upon addition of 5 (Fig. 1, red data) or 10 (Fig. 1, black data) molar equivalents of GTP-bound Rab3a. The observed changes indicate that the C terminus of a-Syn is the primary site of Rab3a interactions and are similar to those previously reported for the interaction of a-Syn with Rab8a (40). Notably, the interaction could only be observed in the absence of salt, suggesting that it is driven largely by electrostatics. Both chemical shift and intensity changes at the C terminus of a-Syn upon addition of GTP-bound Rab3a vanished in the presence of 50 mM NaCl even at molar ratio of 1:11 and 0.5:11 (Fig. S1), although slight intensity decreases were observed for the very N terminus (residues 3–9).

### A-Syn interacts with a positively charged patch on GTP-bound Rab3a that includes the switch-I and switch-II regions

**Figure 2:**
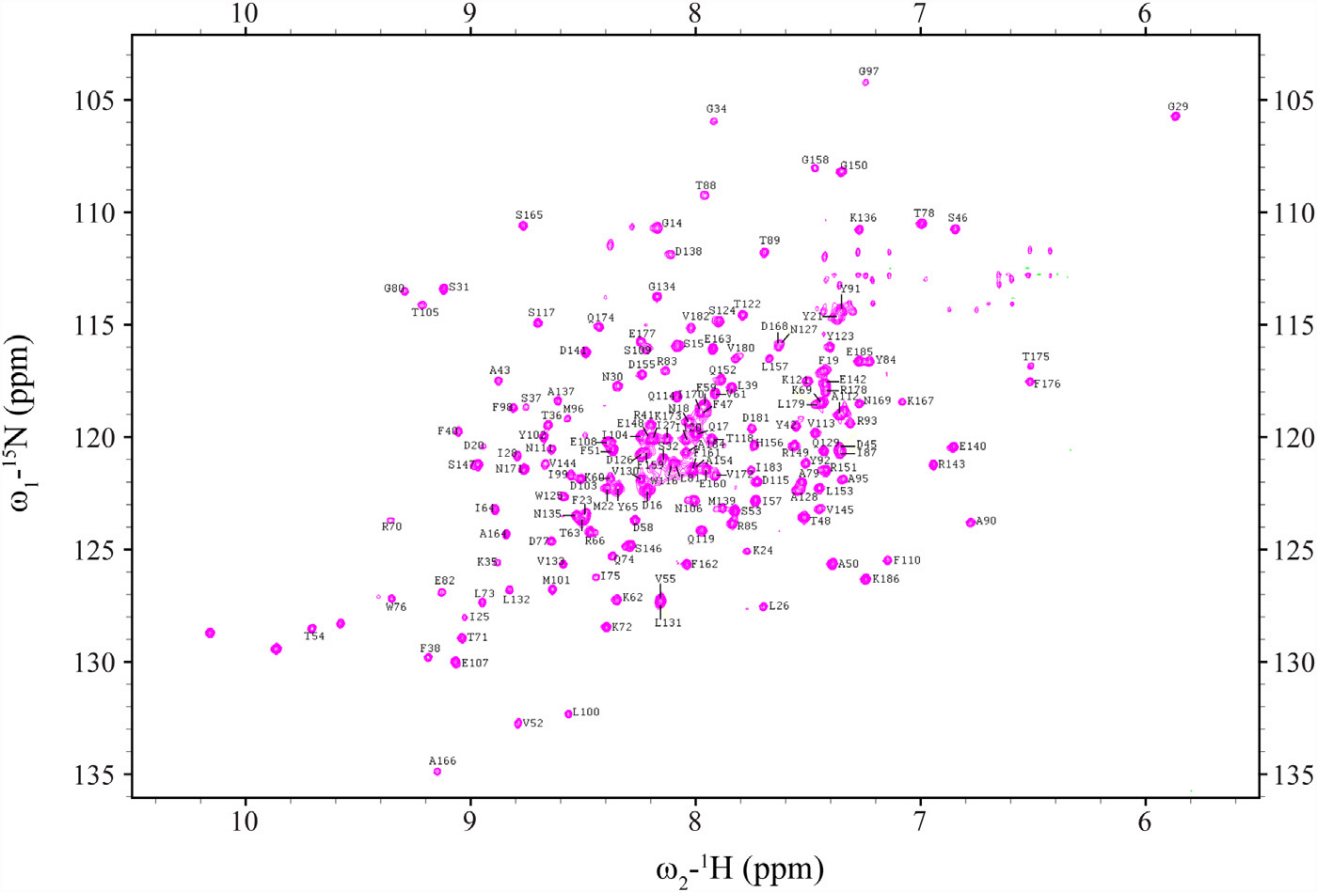
**TROSY spectrum of**^**2**^**H**^**13**^**C**^**15**^**N-labeled GTP-bound Rab3a.** Sequence-specific backbone resonance assignments are indicated.

To explore the binding site for a-Syn on Rab3a, we produced recombinant ^15^N-labeled and ^2^H^13^C^15^N-labeled GTP-bound Rab3a and obtained sequence-specific backbone NMR resonance assignments for the GTP-bound state of the protein. In total, 165 backbone ^1^H, ^13^C, and ^15^N NMR assignments out of 171 nonproline residues were obtained unambiguously (Fig. 2). We then titrated ^15^N-labeled GTP-bound Rab3a with unlabeled a-Syn. However, despite extensive efforts, we were unable to observe reproducible changes in the ^1^H-^15^N HSQC spectrum of Rab3a in the presence of a-Syn even at stoichiometries as high as 20:1 a-Syn:Rab3a. As an alternative approach to identify the Rab3a binding site for a-Syn we turned to paramagnetic relaxation enhancement (PRE) measurements. PRE can establish the physical proximity of different sites within a protein or between proteins in a complex through the degree of attenuation of the NMR signals arising from the presence of a spin-labeled site. We quantified PREs as the intensity ratio of cross-peaks in the ^1^H-^15^N HSQC spectra of a 1-oxyl-2,2,5,5,-tetramethylpyrroline-3-methyl-methanethiosulfonate (MTSL) nitroxide spin-labeled protein, in the absence and presence of ascorbic acid, which was added to the spin-labeled sample to reduce the nitroxide spin label. Residues within ∼20 Å of the spin-labeled site are expected to exhibit attenuated intensities.

**Figure 3:**
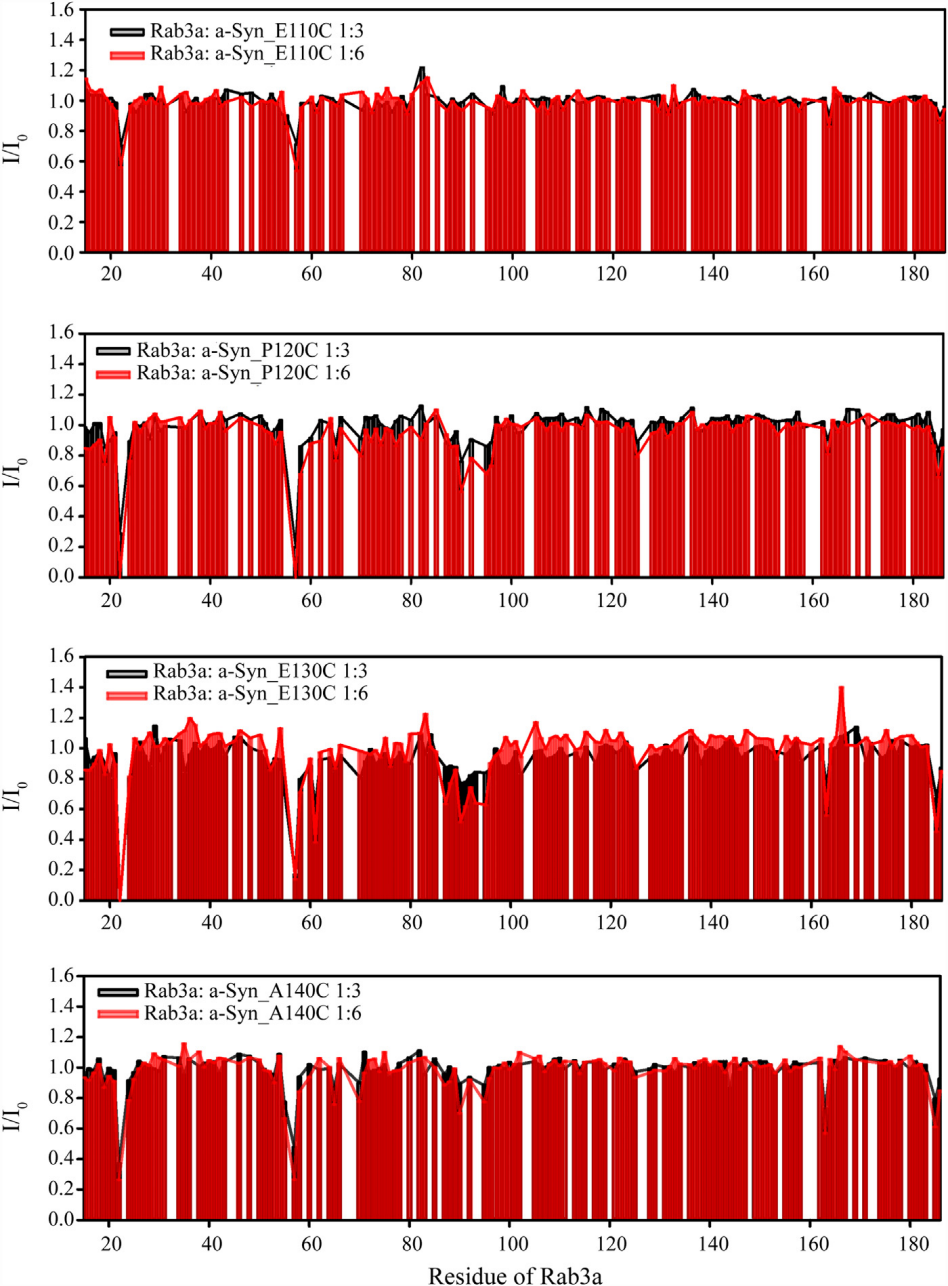
**a-Syn interacts with the switch-I and -II region of GTP-bound Rab3a.** NMR signal intensity ratios for ^15^N-labeled GTP-bound Rab3a in the presence of ^14^N a-Syn_E110C (*top panel*), a-Syn_P120C, a-Syn_E130C or a-Syn_A140C (bottom panel) spin-labeled with MTSL *versus* reduced by ascorbic acid at 1:3 (*black*) and 1:6 (*red*) stoichiometries. Intensity ratios below 1 indicate proximity of the spin labeled position in a-Syn to the affected region of Rab3a. a-Syn, alpha-synuclein; MTSL, 1-oxyl-2,2,5,5,-tetramethylpyrroline-3-methyl-methanethiosulfonate.

**Figure 4:**
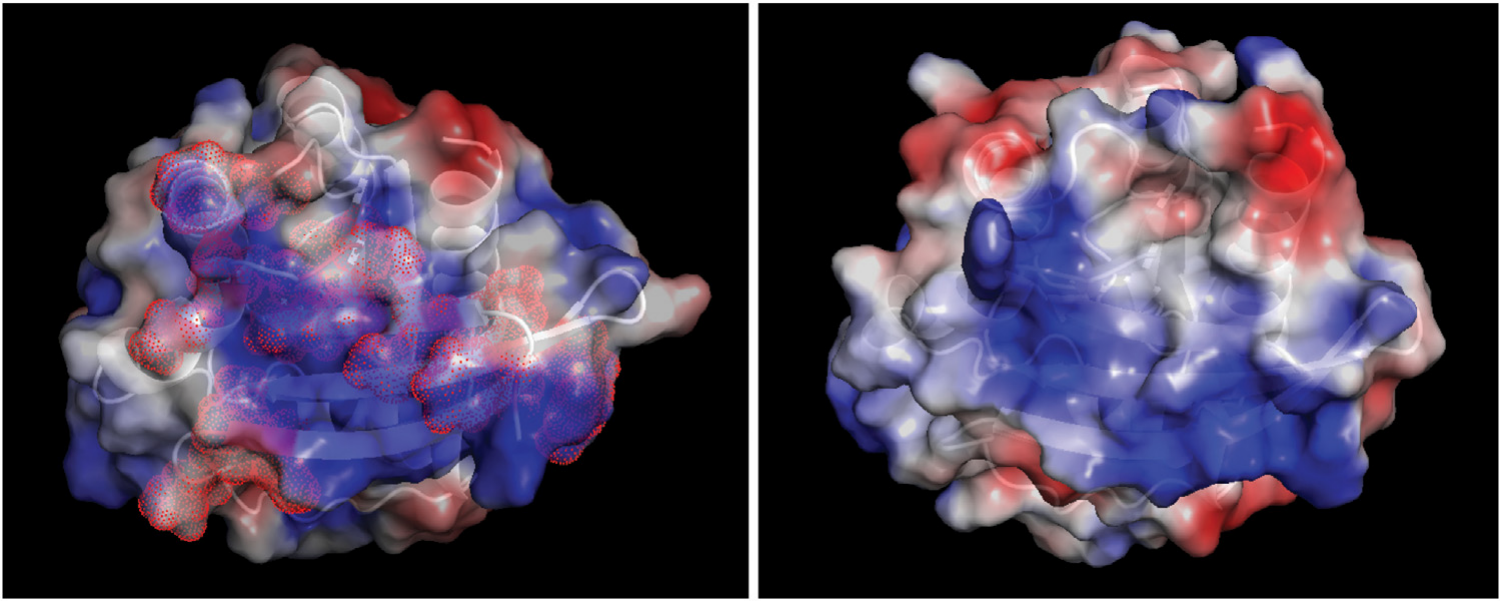
**a-Syn binds to a positive patch on the surface of Rab3a.***Left:* The structure of Rab3a (PDB ID 3RAB residues 18–186 bound to GppNHp) colored to show surface electrostatic potential (calculated using PyMOL) with residues implicated in binding the C-terminal tail of a-Syn shown using a dot surface representation. *Right:* The structure of Rab8a (PDB ID 3QBT, residues 9–178 bound to GppNHp) colored to show surface electrostatic potential. A positive patch that was shown to bind Rab8a (40) is evident and appears similar to the a-Syn binding surface of Rab3a shown in the left panel. a-Syn, alpha-synuclein.

We collected PRE data for ^15^N-labeled GTP-bound Rab3a in the presence of a-Syn spin labeled at four different sites in its C-terminal tail, as this was the a-Syn region that was observed to bind to Rab3a (Fig. 1). Spin labels were introduced by conjugating an MTSL nitroxide group to cysteine residues introduced *via* site directed mutagenesis (E110C, P120C, E130C, and A140C). Importantly, a-Syn contains no naturally occurring Cys residues. PRE effects in GTP-bound Rab3a in the presence of a-Syn spin labeled at different sites within its C-terminal tail (Fig. 3) are observed within the switch-I (residues 49–61, comprising the loop between helix-1 and strand-2 as well as part of strand-2 (38, 41)) and switch-II (residues 76–96, comprising part of strand-3, the following loop, helix-2 and the following short loop (38, 41)) regions, as well as in the vicinity of positions 20, 60 to 70, 125, 163, and 185. More specifically, PREs were observed for residues M22, V55, I57, and E163 (a-Syn_E110C); F19, M22, K24, V55, I57-D58, Y65, R70, A90, Y92, A95-M96, and W125 (a-Syn_P120C); E18, M22, I57, D58, V61, I87-T88, A90-A95, W125, E163, and E185 (a-Syn_E130C); and M22, K24, V55, I57, Y65, R70, A90, A95, E163, and E185 (a-Syn_A140C). Mapping these residues onto the surface of the known structure of Rab3a reveals a positively charged patch (Fig. 4, left), consistent with the high negative charge content of the a-Syn C-terminal tail. Notably, the interface we identify using PREs is more extensive than that previously reported for a-Syn on the surface of Rab8a (40). In that study, binding site residues were largely restricted to helix-2 of switch-II and the G2 loop between helix-1 and strand-2, based on Rab8a chemical shift and intensity changes which, although detectable, were quite noisy. The additional binding regions, beyond helix-2 and the G2 loop that we observe here are likely also involved in a-Syn binding to Rab8a, given that Rab8a features a positively charged surface patch quite similar to that of Rab3a (Fig. 4, right), and that docking studies indicated the involvement of this entire patch in a-Syn binding to Rab8a (40).

### A-Syn-Rab3a interactions are promoted by covalent linkage

**Figure 5:**
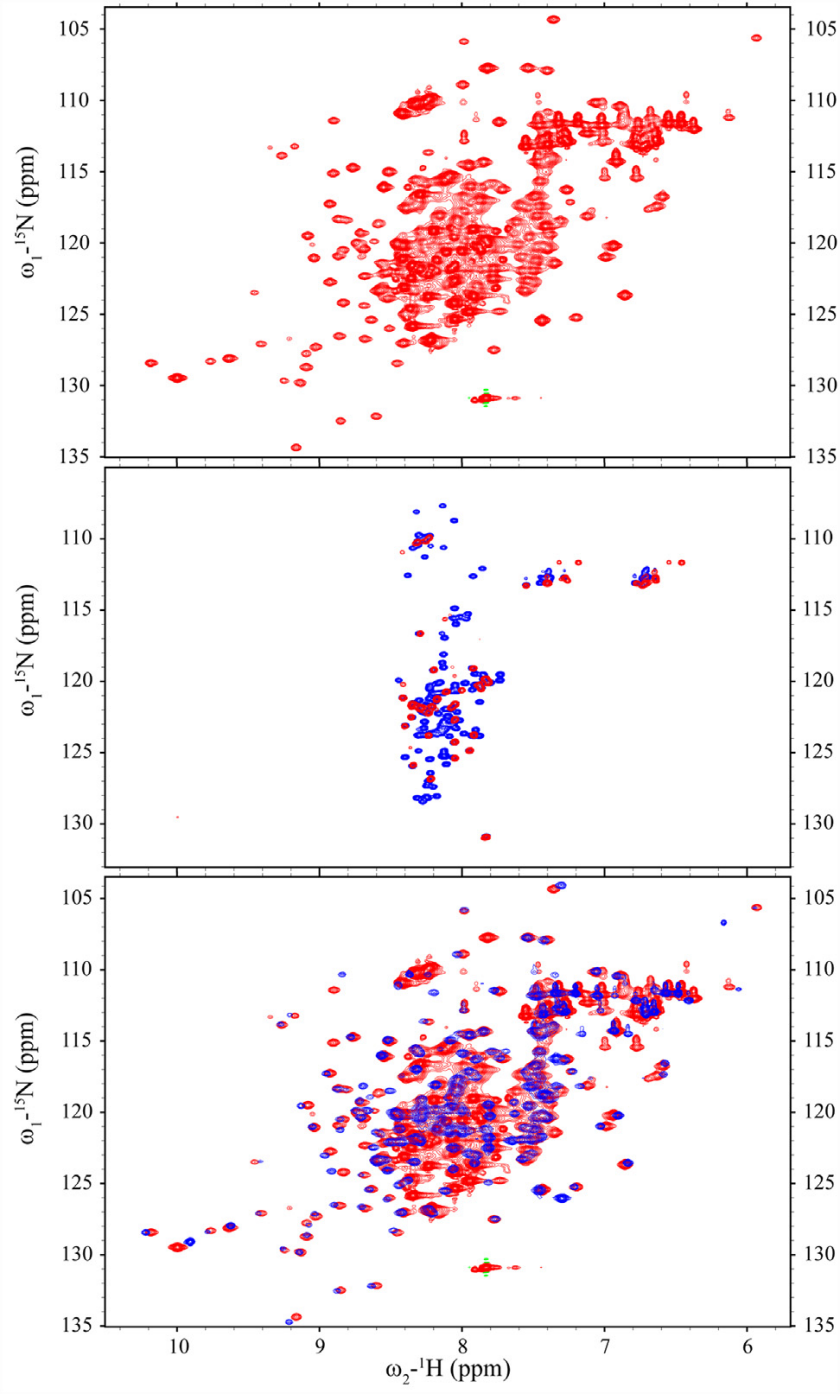
**The C terminus of a-Syn interacts more strongly with Rab3a in the context of a fusion protein.** NMR ^1^H-^15^N HSQC spectra of ^15^N-labeled Rab3a fused to the C-terminus of a-Syn (*top*). Intense narrow signals with limited dispersion that remain visible when the contour level cutoff is raised (*red*) correspond to resonances of free a-Syn in solution (*blue*) that originate from the C terminus of the protein (*middle*). Weaker and more widely dispersed signals (*red*) correspond to resonances from free Rab3a in solution (*blue*) (*bottom*). a-Syn, alpha-synuclein.

**Figure 6:**
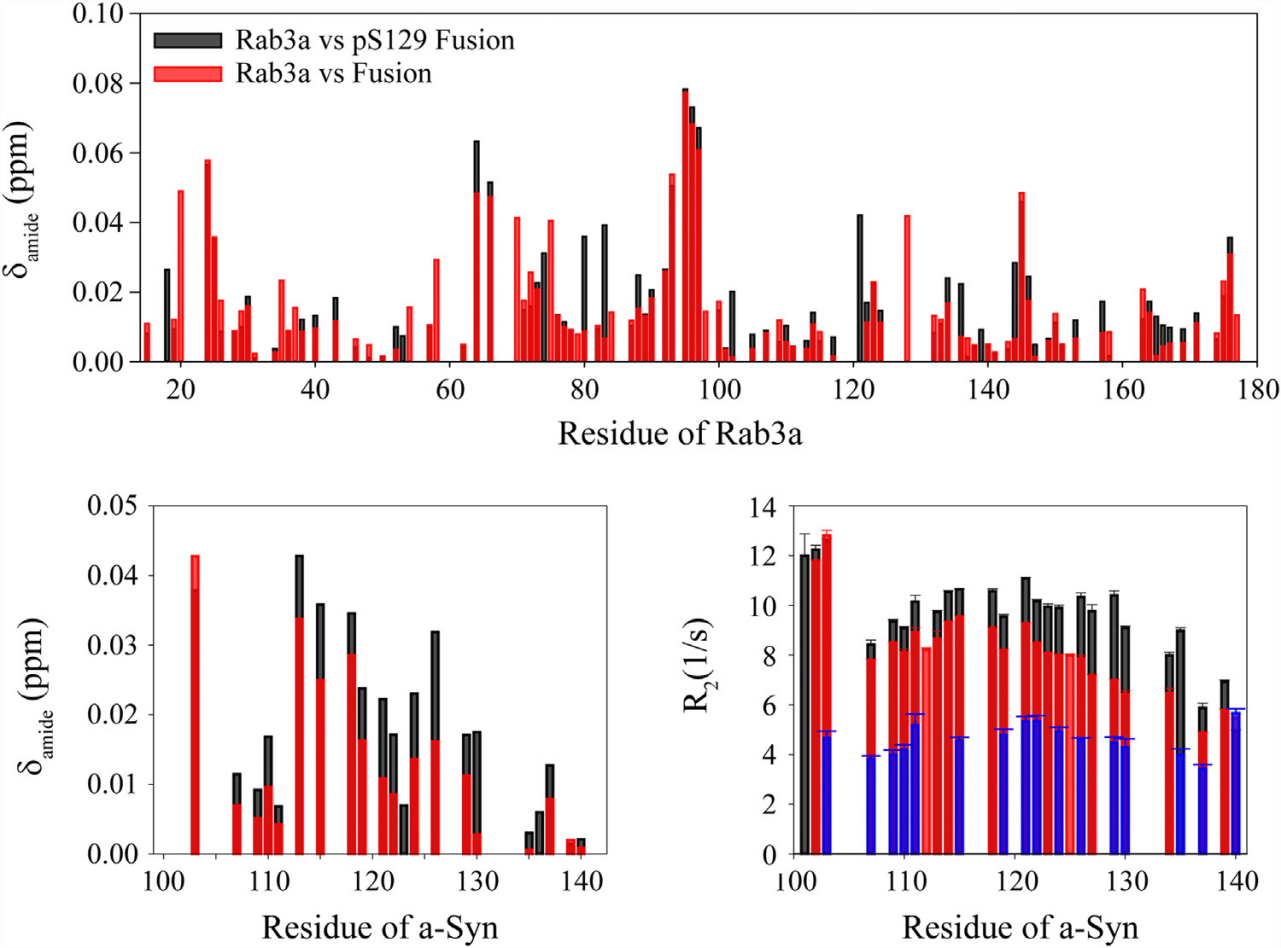
**Rab3a interactions with a-Syn are enhanced in the context of a fusion protein.** (*Top*) Chemical shift changes in Rab3a in the presence of the covalently attached C terminus of a-Syn indicate sites of a-Syn interaction within Rab3a. (*bottom left*) Chemical shift changes in the covalently attached C terminus (*red*) indicate sites of Rab3a interactions. Increased chemical shift changes of the amide cross peak (Δδ_amide_ = √1/2(Δδ_ΗΝ_^2^ + (Δδ_Ν_/5)^2^)) for pS129 a-Syn (*black*) indicate that phosphorylation at a-Syn residue Ser 129 enhances Rab3a-a-Syn interactions. (*bottom right*) Increases in the R_2_ relaxation rates for resonances in the covalently attached C terminus of a-Syn (*red*) compared with the same resonances in the free protein (*blue*) indicate interactions with Rab3a in the fusion construct. Further increases in the R_2_ relaxation rates for pS129 a-Syn (*black*) indicate that phosphorylation at a-Syn residue Ser 129 enhance Rab3a-a-Syn interactions. a-Syn, alpha-synuclein.

Both Rab3a and a-Syn bind to SVs *in vivo*, and confinement to the small surface area of such a vesicle may promote their interactions beyond what we observed in solution. To evaluate the effect of enforced proximity on and to facilitate further characterization of this interaction, we generated a fusion construct of Rab3a (residues 15–186) Q81L and C137A with the C-terminal tail of a-Syn (residues 101–140), using a short GSGS glycine-serine linker (see methods). Note that because a-Syn is a highly disordered protein (2, 42), even a relatively short linker is not expected to restrict the ability of the highly dynamic C-terminal tail of a-Syn to reach and engage its Rab3a interaction site(s). ^1^H-^15^N HSQC spectra of this fusion construct (Fig. 5, top) revealed an intense set of sharp peaks and a broader set of more widely dispersed peaks. The sharp signals overlay well with resonances from the C-terminal tail of a-Syn (Fig. 5, middle) and the broad, widely dispersed peaks overlay with the spectrum of Rab3a (Fig. 5, bottom). This indicates that the Rab3a domain of the fusion protein remains intact and that the C-terminal tail of a-Syn remains highly dynamic. Nevertheless, a detailed comparison of the Rab3a chemical shifts in the context of this fusion with those of isolated Rab3a revealed clear chemical shift changes, presumably arising from interactions between the two tethered protein domains. Resonances that exhibited the largest changes included D20, K24, I64, R66, R70, I75, R93, A95-G97, A128, and V145 (Fig. 6, top), which fall into regions that were also implicated in the PRE data above as sites of interaction between Rab3a and a-Syn. Because Rab3a chemical shift changes could not be observed upon incubation with free a-Syn, even at high stoichiometries, the observation of Rab3a chemical shifts in the fusion construct indicates that the interaction between the two domains is stronger when they are constrained to be near each other, as expected. Small chemical shifts observed in resonances from the a-Syn C-terminal tail in the fusion construct, compared with those for a-Syn alone in solution under the same conditions (Fig. 6, bottom left), were similar to those observed with mixtures of the two individual proteins (Fig. 1).

Further evidence of a-Syn-Rab3a interactions in the context of the fusion construct was obtained from measurements of R_2_ transverse relaxation rates for the sharp signals originating from the a-Syn C-terminal domain. R_2_ is dependent on both overall and internal molecular mobility (43) and changes in R_2_ upon binding provide sensitive probes of molecular interactions. The C-terminal 40 residues of a-Syn exhibit higher R_2_ values in the fusion protein than in isolated full length a-Syn (Fig. 6, bottom right), suggesting that they experience either a slower effective tumbling rate or broadening due to conformational exchange (or both) as a consequence of interactions with the fused Rab3a. Notably, both the chemical shifts and R_2_ values of the a-Syn C-terminal domain remain characteristic of a highly disordered and dynamic polypeptide segment, indicating that the interactions between the C-terminal tail and the Rab3a domain are transient and do not lead to the formation of stable structure by a-Syn.

### A-Syn-Rab3a interactions are promoted on the surface of lipid membranes

**Figure 7:**
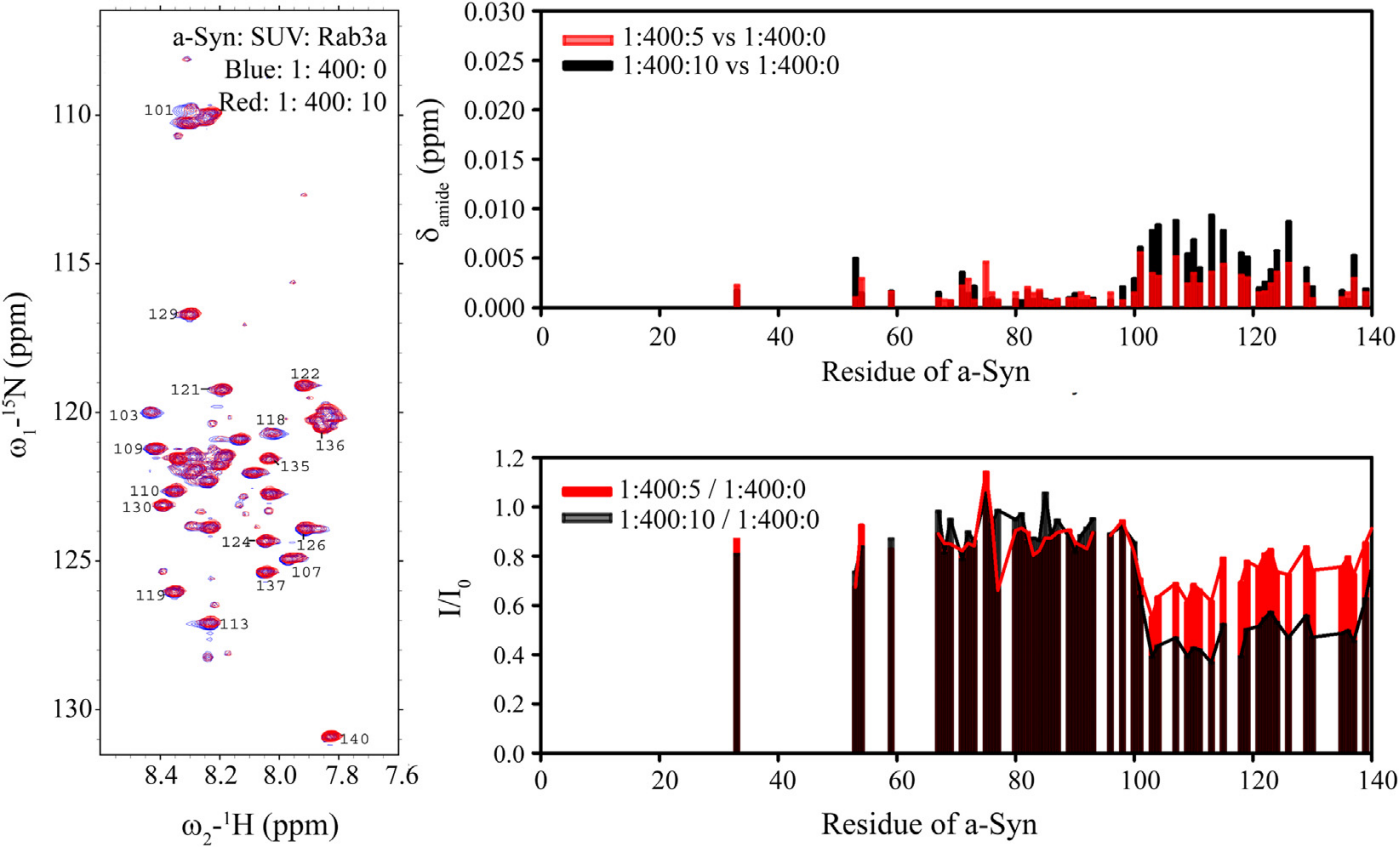
**Rab3a interactions with a-Syn are enhanced on the surface of membranes.** In the absence of Rab3a, the presence of lipid vesicles results in strong attenuation in ^1^H-^15^N HSQC NMR spectra of signals from the N-terminal ∼100 residues of a-Syn, but not of signals from the C-terminal tail of the protein (*left panel*, *blue*). In the presence of 5-fold (*red*) or 10-fold (*black*) excess membrane-localized Rab3a, signals from the a-Syn C-terminal tail (*left panel*, *red*) exhibit both amide chemical shift changes (Δδ_amide_ = √1/2(Δδ_ΗΝ_^2^ + (Δδ_Ν_/5)^2^), *right top panel*) and intensity decreases (*right bottom panel*). Intensity decreases are larger than those observed when the two proteins interact in the absence of membranes (compare Fig. 1). a-Syn, alpha-synuclein; HSQC, heteronuclear single quantum coherence.

Rab3a and a-Syn are both peripheral membrane proteins, and given that covalent linkage of the proteins enhanced their interactions, we considered whether localization to membrane surfaces would have a similar effect. To localize 6-His-tagged Rab3a to membranes, we used SUVs composed of 45: 50: 5 DOPC (1,2-dioleoyl-sn-glycero-3-phosphocholine): DOPS (1,2-dioleoyl-sn-glycero-3-phospho-L-serine): DGS-NiNTA (1,2-dioleoyl-sn-glycero-3-[(N-(5-amino-1-carboxypentyl)iminodiacetic acid)succinyl]). 1D NMR experiments (not shown) confirmed binding of Rab3a to the vesicles. NMR resonances in the lipid-binding domain of a-Syn, comprising the first ∼100 residues of the protein, exhibit decreased intensities in the presence of lipid vesicles, whereas residues in the C-terminal 40 residues of the protein are largely unaffected since this region does not interact strongly with membranes (44, 45). We compared the intensities of a-Syn ^1^H-^15^N HSQC NMR resonances in the presence of a 400:1 ratio of lipids in the form of SUVs with and without a 5- or 10-fold excess of vesicle-bound Rab3a (Fig. 7). Resonances intensities in the C-terminal tail of a-Syn decreased by approximately 25% or 50% at the two stoichiometries (Fig. 7), compared to much smaller decreases of a few percent for the same stoichiometries in the absence of SUVs (Fig. 1). These results indicate that colocalization of a-Syn and Rab3a to membrane surfaces significantly enhances the interaction between the proteins, consistent with the stronger interaction observed when the proteins are covalently linked and as might be expected based on reduced dimensionality and resultant higher effective concentrations of the proteins on the membrane surface.

### Phosphorylation at Ser 129 of a-Syn weakly enhances interactions with Rab3a in solution, but not on membranes

Phosphorylation of a-Syn at residue Ser 129 is strongly associated with pathological a-Syn deposits in disease (16), but the physiological role of this modification is unknown. We generated pS129 a-Syn by coexpressing the protein with the polo-like kinase family member polo-like kinase 2 (PLK2) and examined its interactions with Rab3a. In solution, addition of a 10-fold excess of unlabeled Rab3a to ^15^N-labeled pS129 a-Syn resulted in only a very slight increase in resonance attenuation in the C-terminal tail region compared to changes observed for WT a-Syn (Fig. S2) and no detectable changes in intermolecular PREs (Fig. S3). However, the fusion of Rab3a to the C-terminal tail of pS129 a-Syn resulted in a small but clear increase of chemical shift changes for both a-Syn and Rab3a, as well as in the R_2_ relaxation rates of the a-Syn C-terminal residues (Fig. 6), compared to a fusion with the unmodified C-terminal tail, indicating that in the context of the fusion construct, phosphorylation of a-Syn at residue Ser 129 strengthens the interaction of a-Syn and Rab3a. Surprisingly, in the presence of SUVs, Ser 129 phosphorylation was found to result in slight decrease of membrane- and Rab3a-associated a-Syn (Fig. S4), as indicated by higher intensity ratios both in the N-terminal lipid-binding domain and in the C-terminal tail compared with unmodified a-Syn, possibly as a result of charge repulsion between the phosphate group and the negatively charged phospholipid headgroups.

### GTPase activity of Rab3a is inhibited by a-Syn in solution and on membranes

**Figure 8:**
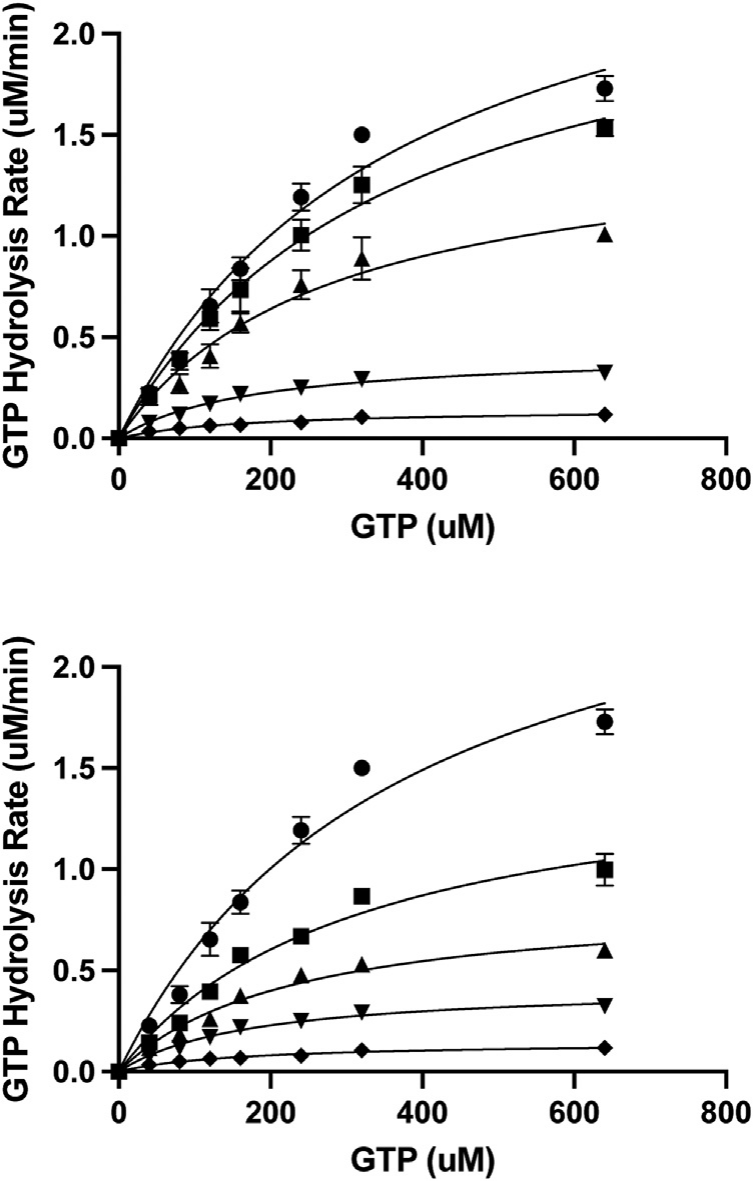
**a-Syn inhibits the GTPase activity of Rab3a.** GTP hydrolysis rates of Rab3a containing the wildtype Q residue at position 81, measured by phosphate release as a function of time at different GTP concentrations, in the absence (*circles*) or presence of 1:1 (*upper panel*) or 10:1 (*lower panel*) a-Syn (*squares*) or pS129 a-Syn (*triangles*) or for the fusion of the a-Syn C-terminal tail with Rab3a (*inverted triangles*) or for GTPase deficient Q81L Rab3a (*diamonds*). Measurements were performed at 37 °C at a final Rab3a concentration of 0.5 μM. *Solid lines* represent nonlinear least squares fits of the data to the Michaelis–Menten equation. The same data are shown for the fusion construct and the Q81L mutant in both the upper and lower panels. Error bars represent the standard deviation of three independent measurements. a-Syn, alpha-synuclein.

**Figure 9:**
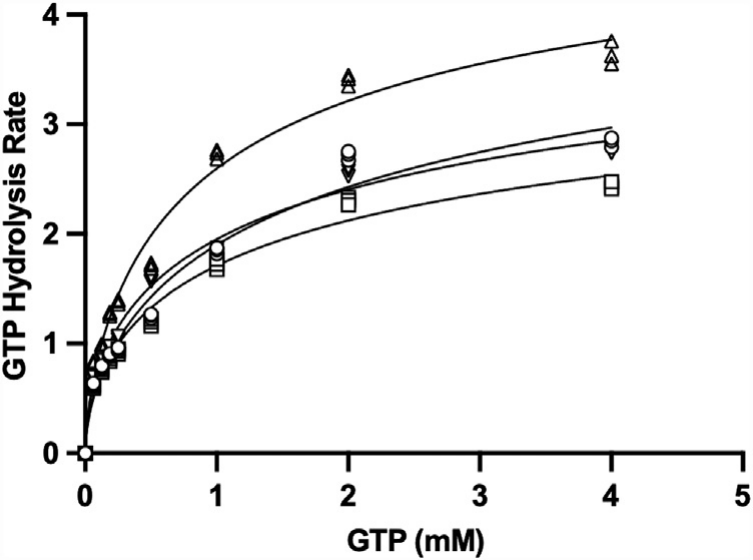
**a-Syn inhibits the GTPase activity of C-terminally SUV-anchored Rab3a more potently than in solution.** GTP hydrolysis rates of C-terminally His-tagged Rab3a measured by phosphate release as a function of time at different GTP concentrations, in solution in the absence (*circles*) or presence (*squares*) of 10:1 a-Syn or anchored to SUVs *via* the C-terminal His-tag in the absence (*triangles*) or presence (*inverted triangles*) of 10:1 a-Syn. Measurements were performed at 37 °C at a final Rab3a concentration of 0.5 μM. *Solid lines* represent nonlinear least squares fits of the data to the Michaelis–Menten equation. Error bars represent the standard deviation of three independent measurements. a-Syn, alpha-synuclein; SUV, small unilamellar vesicle.

The binding site for a-Syn on Rab3a mapped by PREs and chemical shift changes closely resembles the binding site for the effector domain of the Rab3a effector rabphilin-3A (41). Because rabphilin-3A alters the enzymatic activity of Rab3a (46), acting as a weak stimulator of basal GTPase activity but as an inhibitor of Rab3A GAP-stimulated activity, we examined the effect of a-Syn and pS129 a-Syn on GTP hydrolysis by Rab3a. Using a simple colorimetric assay for GTP hydrolysis (47), we found that the addition of WT or pS129 a-Syn inhibits GTP hydrolysis of Rab3a in a concentration-dependent manner, with pS129 a-Syn exhibiting a stronger effect (Fig. 8) consistent with our results indicating that Ser 129 phosphorylation enhances a-Syn binding to Rab3a. Measurements of Rab3a activity in the context of the fusion construct with the a-Syn CTD showed that covalent linkage of the proteins enhanced the ability of the a-Syn C-terminal tail to inhibit Rab3a activity. Fitting of data obtained at increasing substrate concentrations to the Michaelis Menton equation reveals that the presence of a-Syn results in a decrease in Vmax, but also in a decrease in Km (Table 1), suggesting that the protein may act as an uncompetitive inhibitor. Consistent with this possibility, the slopes of the Lineweaver-Burke plots remained roughly constant in the presence or absence of a-Syn (Fig. S5). Unexpectedly, the presence of SUVs resulted in a decreased activity of Rab3a (Fig. S6), suggesting that anchoring of this Rab3a construct to vesicles *via* its N-terminal 6-His-tag interferes with its catalytic activity. Indeed, the activity of SUV-anchored Rab3a was comparable to that observed for Rab3a in solution in the presence of 10-fold a-Syn, and addition of 1× or 10× a-Syn did not appreciably decrease the measured activity of the SUV-anchored protein further (not shown). Since Rab3a is anchored to membranes *via* prenylation of its C-terminal tail, we explored whether moving the His-tag to the C terminus of Rab3a would restore the activity of the SUV-anchored protein. Indeed, this construct resulted in an enhancement of Rab3a GTPase activity when anchored to SUVs (Fig. 9). Addition of a-Syn to this construct resulted in inhibition of enzymatic activity both in solution and in the presence of SUVs, but the effect was much greater in the presence of SUVs (Fig. 9 and Table 2), consistent with our observations of increased Rab3a-a-Syn interactions on the membrane surface and with our hypothesis that this interaction is physiologically relevant. We do note that the degree of inhibition of the C-terminally tagged Rab3a construct in solution was smaller than that observed at comparable stoichiometry for the N-terminally tagged construct (Fig. 8, bottom v*ersus* Fig. 9). This could potentially result from the different position or the greater positive charge of the C-terminal 10-His-tag, which could perhaps screen the positively charged binding patch on the surface of Rab3a. Future studies including the intact CTD of Rab3a and with a more physiological lipid anchor will likely be required to conclusively resolve this issue.

**Table 1:** Km and Vmax values for Rab3a in the absence or presence of WT or pS129 a-Syn or in the context of the Rab3a-a-Syn fusion construct

Preparation	Km	Vmax
Rab3a	376 ± 89 μM	2.9 ± 0.5 μM/min
Rab3a: WT a-Syn = 1:1	369 ± 73 μM	2.5 ± 0.3 μM/min
Rab3a: pS129 a-Syn = 1:1	273 ± 71 μM	1.5 ± 0.3 μM/min
Rab3a: WT a-Syn = 1:10	316 ± 40 μM	1.6 ± 0.2 μM/min
Rab3a: pS129 a-Syn = 1:10	230 ± 37 μM	0.86 ± 0.10 μM/min
Rab3a-a-Syn	174 ± 46 μM	0.43 ± 0.04 μM/min

**Table 2:** Km and Vmax values for C-terminally His-tagged Rab3a in solution or when anchored to SUVs in the absence or presence of 10× a-Syn

Preparation	Km	Vmax
Rab3a	438 ± 63 μM	2.8 ± 0.5 μM/min
Rab3a: WT a-Syn	380 ± 59 μM	2.5 ± 0.9 μM/min
Rab3a: SUV	510 ± 52 μM	4.0 ± 0.5 μM/min
Rab3a: SUV: WT a-Syn	383 ± 36 μM	2.9 ± 0.6 μM/min

## Discussion

Despite intense interest in a-Syn in the context of its aggregation and deposition in PD and related synucleinopathies, the normal function(s) of a-Syn remain poorly understood (48, 49, 50, 51, 52). Beginning with its original identification as a protein enriched in synaptosome preparations from electric eels (53), a substantial body of work has implicated a-Syn as playing a functional role in the SV cycle (5, 11, 51, 54, 55). Nevertheless, mechanistic insights into how a-Syn could influence SV trafficking and release have remained sparse and have largely been confined to investigations of a-Syn interactions with SNARE proteins and its effects on SNARE-mediated vesicle fusion (6, 56, 57, 58), including fusion pore formation (59, 60). More recent work has extended this focus to the area of SV cluster organization and the potential roles of a-Syn as a modulator of liquid–liquid phase separation processes at presynaptic nerve terminals (12, 61). Studies of pathways influenced by a-Syn in living cells have highlighted both functional and pathological interactions between a-Syn and Rab proteins (8, 27, 33, 62, 63, 64, 65), which act as master regulators of vesicle trafficking pathways in eukaryotic cells (66). Functional interactions of a-Syn specifically with Rab3a, the primary Rab regulating SV trafficking (67), have also been reported (27, 33, 34, 68). Direct but weak interactions of a-Syn with a different neuronal Rab, Rab8a, were characterized using NMR spectroscopy (40) and demonstrated that the negatively charged C-terminal tail of a-Syn binds to a positive patch on the surface of Rab8a which is formed in part by the switch-I and switch-II regions. This interaction was shown to influence the aggregation of a-Syn, but its functional consequences were not explored.

Here, we have undertaken a study of the interactions between a-Syn and Rab3a, the master regulator of SV trafficking in neurons. We hypothesized that such interactions play a role in the normal function of a-Syn and may contribute to its regulation of the SV pathway. Using NMR, we showed that in solution, the highly negatively charged C-terminal tail of a-Syn binds weakly to a positive patch on the surface of Rab3a in a manner analogous to its interaction with Rab8a, despite the limited sequence homology between these proteins. This interaction is likely driven at least in part by electrostatics, since was disrupted in the presence of salt (Fig. S1). The weakness of the interaction and its salt dependence may both call into question its physiological relevance. However, because both a-Syn and Rab3a are expected to colocalize on the surface of SVs, at a higher effective concentration than in solution, we explored whether artificially tethering the C terminus of a-Syn to Rab3a would result in stronger interactions. NMR chemical shift perturbations and relaxation measurements confirm that binding between the two proteins is enhanced in the context of this fusion construct. We further explored whether membranes would potentiate the interactions of the proteins and found that the presence of small unilamellar lipid vesicles substantially increased the binding of a-Syn to Rab3a. This result is consistent with previous data indicating that a-Syn and Rab3a form a complex on cellular membranes but not in the cytosol (34) and support the notion that their interaction may be physiologically relevant.

To explore the functional consequences of this interaction, we noted that the binding site of a-Syn on Rab3a is highly similar to the binding site occupied by the Rab3a effector rabphilin-3A, which has been reported to alter the GTPase activity of Rab3a by stimulating basal activity but inhibiting GAP-stimulated activity (41). We therefore assayed the effects of a-Syn on the GTP hydrolysis activity of Rab3a *in vitro*. We demonstrated that a-Syn inhibits the activity of Rab3a by decreasing Vmax, possibly acting as an uncompetitive inhibitor, and that this effect is strongly enhanced in the context of the fusion construct between a-Syn and Rab3a. Anchoring Rab3a to SUVs using an N-terminal 6-His-tag resulted in decreased GTPase activity that was no longer sensitive to a-Syn. This suggests that this anchoring mode, in contrast with the natural anchoring *via* geranyl-geranyl groups at the end of the C-terminal hypervariable region, interferes with the activity of Rab3a. Moving the His-tag to the C terminus of Rab3a resulted in a construct that exhibits increased GTPase activity when anchored to SUVs. Inhibition of this activity by a-Syn is significantly stronger when Rab3a is anchored to SUVs than when it is free in solution (Fig. 9 and Table 2), consistent with our observations of enhanced Rab3a binding to a-Syn on the surface of SUVs. However, fully recapitulating the effects of a-Syn on the activity of membrane-anchored Rab3a will likely require assays performed with authentically prenylated Rab3a, a challenging future endeavor that is being pursued.

Phosphorylation of a-Syn at residue Ser 129 is mediated by the polo-like kinase family (69, 70) and is closely associated with pathological deposition of a-Syn in PD (16). Polo-like kinases have been implicated in the regulation of synaptic plasticity (71, 72, 73), but a specific role for PLK phosphorylation of a-Syn Ser 129 has not been reported to date, although a-Syn is also a known regulator of synaptic plasticity (74). Thus, the functional consequences of this PTM remain unknown, but its location in the C-terminal tail of the protein suggests that it could modulate intermolecular interactions involving this region. Ser 129 phosphorylation was previously reported to enhance a-Syn binding to Rab8a in solution (40), and we observe a similar enhancement of Rab3a binding here. This effect is also evident in the fusion construct between the two proteins, but surprisingly not in the presence of phospholipid vesicles, possibly as a result of decreased synuclein-membrane interactions, as was previously reported to result upon nitration of tyrosine residues in the C-terminal tail of a-Syn (75). Ser 129 phosphorylation lead to an increased inhibition of Rab3a activity by a-Syn in solution and in the context of the fusion construct, indicating a potential role for this PTM in modifying this activity of a-Syn and further implicating this activity as a physiological function of the protein *in vivo*.

## Conclusions

Our results reveal that a-Syn binds to Rab3a in a manner similar to that by which it engages with Rab8a but that this interaction is potentiated when the two proteins are bound to the surface of lipid vesicles resembling SVs in size. Furthermore, we show that a-Syn binding to Rab3a results in a decreased GTP hydrolyzing activity and that this inhibition is enhanced by phosphorylation of a-Syn at residue Ser 129, a modification tightly correlated with a-Syn pathology in disease. These observations suggest that a-Syn may function in part by binding to and regulating the activity of Rab3a on SVs, which in turn would be expected to alter their trafficking and release. This conclusion is consistent with previous reports of functional interactions between a-Syn and Rab proteins but provides new mechanistic insights into the basis of the interaction and its functional consequences.

## Experimental procedures

### Protein expression and purification

Recombinant WT a-Syn, a-Syn_E110C, a-Syn_P120C, a-Syn_E130C, and a-Syn_A140C were produced as previously reported (76). Briefly, for protein uniformly labeled with ^15^N for NMR titrations or ^14^N for PRE experiments, *Escherichia coli* BL21 (DE3) cells were transformed with a plasmid encoding a-Syn and were grown at 37 °C in minimum media containing ^15^N ammonium chloride or ^14^N ammonium sulfate as the sole nitrogen source. Protein expression was induced with isopropyl 1-thio-β-d-galactopyranoside at an absorbance of ∼0.6, for 3 to 4 h at 37 °C before harvesting by centrifugation. Purification of a-Syn proceeded as previously reported (77). The purified protein was lyophilized and stored at −80 °C.

For the expression and purification of phosphorylated a-Syn, a-Syn_E110C, a-Syn_P120C, a-Syn_E130C, and a-Syn_A140C at Ser 129, *E. coli* BL21 (DE3) cells were cotransformed with plasmids encoding a-Syn and PLK2. Quantitative phosphorylation at a-Syn residue Ser 129 was verified by 2D ^1^H-^15^N HSQC NMR spectra for isotopically labeled protein.

A construct encompassing the GTPase domain of Rab3a (residues 15–186) preceded by an N-terminal 6-His-tag peptide in a modified pET15b vector (41) was modified by site-directed mutagenesis to incorporate the C137A and/or Q81L mutations and then used to express Rab3a in *E. coli*. BL21(DE3) cells transformed with the plasmid were grown at 37 °C in either LB media or minimum media containing ^15^N ammonium chloride as the sole nitrogen source (for ^15^N-labeled protein) or ^15^N ammonium chloride and ^13^C Glucose as the sole nitrogen and carbon source in 100% D_2_O (for triply-labeled ^2^H^13^C^15^N protein for backbone resonance assignments). Protein expression was induced at an absorbance of ∼0.6 by addition of 1 mM isopropyl 1-thio-β-d-galactopyranoside, and cells were harvested after an additional 4 h at 28 °C. Cell pellets were resuspended in 50 mM Tris, pH 8.0, ± DTT, lysed by sonication, centrifuged at 40k rpm for 1 h, and the supernatant was loaded onto a Ni-NTA-agarose column (Qiagen). After washing with 2 column volumes of 50 mM Tris, pH 8.0, 500 mM NaCl, 10 mM imidazole, ± DTT, the fusion protein was eluted with a gradient of 20, 50, 100, and 150 mM imidazole. For NMR, fractions containing Rab3a were confirmed by sodium dodecyl sulfate polyacrylamide gel electrophoresis and were dialyzed against 50 mM Tris, pH 8.0, 10 mM MgCl_2_ at least 3 times. 1 mM GTP were further added after the dialysis to obtain the GTP-bound form of Rab3a, and pH was adjusted to ∼7.1. For GTPase activity assay, fractions eluted with 150 mM imidazole were dialyzed against 20 mM Hepes-NaOH, pH 7.5 at least three times snap-frozen in liquid nitrogen and stored at −80 °C. Protein concentration was determined using a NanoDrop spectrophotometer (ThermoFisher Scientific) by UV absorbance at 280 nm with an extinction coefficient of 28,420 M^-1^ cm^-1^ and MW of 21.0 kDa. To produce C-terminally His-tagged Rab3a the N-terminal 6-His-tag of the Rab3a construct described above was deleted, and a 10-His-tag was inserted after the C-terminal residue. The deletion and insertion were accomplished using the Q5 site-directed mutagenesis protocol (New England Biolabs).

A fusion construct of Rab3a Q81L and C137A residues 15 to 186 to residues 101 to 140 of a-Syn, linked by a GSGS linker, was constructed by using PCR amplification to introduce BamHI and EcoRI restriction sites before and after Rab3a and an EcoRI restriction site overlaid onto a GSGS coding sequence before a-Syn residue 101 with a SalI restriction site after a-Syn residue 140. A three-piece T4 ligase reaction of the empty BamHI/SalI digested pET15b plasmid, the BamHI/EcoRI digested Rab3a fragment, and the EcoRI/SalI digested GSGS-a-Syn101-140 fragment resulted in a new pET15b plasmid containing the desired fusion with the residual EcoRI site. Site-directed mutagenesis was used to correct the EcoRI site to the desired GSGS coding sequence, generating 6-His-tagged Rab3a Q81L 15-186 linked by a GSGS linker to a-Syn 101-140 in the pET15b vector, as confirmed by DNA sequencing. Site-directed mutagenesis was used to restore the wildtype Q residue at position 81 in the fusion construct used for activity assays. Expression and purification and storage of the fusion construct followed the protocols described above for Rab3a and S129 phosphorylation was accomplished by co-transfection with PLK2 as described above.

### Preparation of SUVs

All lipid vesicle stock solutions were freshly prepared, and all samples containing lipid vesicles were kept at 4 °C. Lipid vesicles (DOPC: DOPS: DGS-NiNTA 45: 50: 5) were prepared by drying mixtures of different lipids dissolved in chloroform (Avanti Polar Lipids) under nitrogen gas using a SpeedVac. The lipid mixture was resuspended using corresponding buffer (50 mM Tris, pH 8.0, 10 mM MgCl_2,_ 10% D_2_O, 1 mM GTP for NMR titrations, and 20 mM Hepes-KOH, pH 7.5 for GTPase), immersed in a bath sonicator for 30 min, and clarified by ultracentrifugation at 130,000*g* for 2 h as modified from Bussell and Eliezer (44). The supernatant was used as SUV stock solution.

### NMR titrations and resonance assignment

NMR experiments were performed on 600-MHz Bruker Avance, 800-MHz Bruker Avance, or 900-MHz Bruker Avance spectrometers equipped with cryogenic probes. Two-dimensional ^1^H,^15^N-HSQC experiments were acquired for titrations, with a typical spectral width of 25 ppm in the ^15^N dimension and 20 ppm in the ^1^H dimension. Lyophilized ^15^N-labeled WT or pS129 a-Syn was dissolved in NMR buffer (50 mM Tris, 10 mM MgCl_2_, 10% D_2_O, 1 mM GTP, pH7.1) and centrifuged (25,000 RCF) to remove large molecular weight aggregates. Titration samples were prepared by mixing stock solutions of ^15^N-labeled WT or pS129 a-Syn: SUV: unlabeled Rab3a to achieve the desired solute concentrations as following: 1:0:0, 1:0:5, 1:0:10, 1:400:0, 1:400:5, and 1:400:10. To make direct comparison of ^15^N-labeled a-Syn and pS129 a-Syn, the concentrations were first assayed using UV absorbance and then further confirmed by 1D NMR. In order to monitor the effect of NaCl on a-Syn-Rab3a interaction, samples of ^15^N-labeled WT a-Syn: unlabeled Rab3a 1:0, 1:11, and 0.5:11 were prepared for ^1^H,^15^N-HSQC titration, using a modified NMR buffer of 50 mM Tris, 50 mM NaCl, 10 mM MgCl_2_, 10% D2O, 1 mM GTP, 0.05% NaN_3_, pH7.1. All spectra were measured at 10 °C. Protein concentrations were typically ∼50 μM for ^15^N-labeled a-Syn or pS129 a-Syn.

Two-dimensional TROSY spectra and three-dimensional HNCA, HNCO, HNCACO, HNCACB, HNCOCACB experiments of ^2^H^13^C^15^N-labeled GTP-bound Rab3a were acquired on 600-, 800-, or 900-MHz Bruker Avance spectrometers equipped with cryogenic probes, with a typical spectral width of 25 ppm in the ^15^N dimension and 20 ppm in the ^1^H dimension. Purified ^2^H^13^C^15^N-labeled GTP-bound Rab3a was dissolved in NMR buffer (50 mM Tris, 150 mM NaCl, 2 mM DTT, 10 mM MgCl_2_, 1 mM GTP, 0.05% NaN_3_, 10% D_2_O, pH7.1) at a concentration of ∼260 μM, and all spectra were measured at 10 °C. Spectra were processed with NMRPipe (78) and analyzed with SPARKY (79). Resonance assignments of Rab3a ^13^Cα, ^13^Cβ, ^15^N, and ^1^HN nuclei were obtained manually using the triple resonance data.

### Paramagnetic relaxation enhancement

WT or pS129 a-Syn E110C, P120C, E130C, and A140C mutants were used for PRE measurements (WT a-Syn contains no Cys residues). The ^14^N-labeled proteins were spin labeled with MTSL (Toronto Research Chemicals) as described (80). Briefly, WT or pS129 a-Syn single-cys mutants in 50 mM Tris, 10 mM MgCl_2_, 10% D_2_O, 1 mM GTP, pH 7.1 were incubated with ca. 20-fold molar excess MTSL (prepared in 100% DMSO) for 1 h at 37 °C (900 rpm). To remove free MTSL, the mixture was then dialyzed overnight against ∼55 mM Tris, pH 8.0, 11 mM MgCl_2_. 10% D_2_O and 1 mM GTP were further added after the dialysis, and pH was adjusted to ∼7.1. PRE was measured by collecting ^1^H-^15^N HSQC spectra using ∼150 μM ^15^N-labeled Rab3a: ^14^N spin labeled WT or pS129 a-Syn variants (1:3 and 1:6) at 10 °C. For control diamagnetic samples, ascorbic acid (∼15-fold molar excess in relation to the protein) was added to the MTSL spin-labeled sample to reduce the nitroxide spin label. The intensities of cross-peaks in the ^1^H-^15^N HSQC spectra of both the spin-labeled and reduced samples were measured, and their ratio was calculated.

### R_2_ relaxation

R_2_ relaxation rates were measured through a series of 11 ^1^H-^15^N HSQC-based T_2_ experiments with relaxation time delays of 0, 16, 32, 48, 64, 80, 96, 112, 128, 32, and 32 ms. The intensity decay of each cross-peak was fit to a single exponential decay function using Sparky. All ^1^H-^15^N HSQC spectra and R_2_ relaxation experiments were acquired at 10 °C, using the same NMR buffer (50 mM Tris, 10 mM MgCl_2_, 10% D_2_O, 1 mM GTP, pH 7.1) at protein concentration of ca. 360 μM. Equal protein concentrations in different samples were confirmed using 1D ^1^H NMR. NMR relaxation data were processed with TOPSPIN3.2 (Bruker) and analyzed with SPARKY (79).

### Rab3a GTPase activity measurements

GTP hydrolysis of a Rab3a construct containing the wildtype Q residue at position 81, as well as the C137A mutation, was measured using a colorimetric assay to detect released phosphate as previously reported (47). GTP (Roche Diagnostics GmbH) was dissolved in 20 mM Hepes-NaOH, pH 7.5 and then diluted at different concentrations in GTPase assay buffer containing 20 mM Hepes-NaOH, pH 7.5, 2 mM MgCl_2_, and 1 mM DTT. Proteins and SUVs were prepared as described above in 20 mM Hepes-NaOH, pH 7.5. GTP hydrolysis in the absence and presence of 200 μM SUV lipids and of 0.5 μM or 5 μM of WT or pS129 a-Syn was initiated by adding protein mixtures to GTP solutions in assay buffer at a final Rab3a concentration of 0.5 μM and incubating at 37 ^°^C. At each time point, 20 μl of the reaction mixture (400 μl initial volume) was transferred to a 96-well flat bottom plate containing 5 μl of 0.5 M EDTA, pH 8.0 to stop the reaction. 150 μl of Malachite Green stock solution (1 mM Malachite Green, 10 mM ammonium molybdate in 1N HCl, filtered through a 0.2 μM Millipore membrane and covered with foil to avoid light exposure) was added to each reaction, and absorbance at 650 nm was measured using a SpectraMax M5 plate reader (Global Headquarters, LLC). All measurements were performed in triplicate. A standard curve using 10 to 100 μM Pi was measured in parallel for each experiment. Absorbance of buffer only ± GTP was measured as a subtraction blank. Reaction rates were obtained as linear fits to early time points, and the Michaelis constant (*K*m) and the maximal reaction velocity (*V*max) for Rab3a GTPase hydrolysis were obtained from nonlinear least squares fits of the reaction rates as a function of GTP concentration to the Michaelis Menten equation using Prism 9 software (GraphPad). Controls using the Q81L mutant, which is deficient in GTP hydrolysis, were performed in the same manner. The activity of the fusion construct of Rab3a with residues 101 to 140 of a-Syn, containing C137A and the wildtype Q residue at position 81, was measured in the same manner.

## Data availability

NMR chemical shift assignments for GTP-bound Rab3a have been deposited in the BMRB database (BMRB accession number 51380). All other data are available upon request to David Eliezer (dae2005@med.conell.edu).

## Supporting information

This article contains supporting information.

## Conflicts of interest

The authors declare that they have no conflicts of interest with the contents of this article.

## Supporting information

Supporting figures S1−S6
