# Supplementary material for: Molecular and functional interactions of alpha-synuclein with Rab3a
Source: J Biol Chem. 2022 Jul 6;298(9):102239. doi: 10.1016/j.jbc.2022.102239 (PMC9396396; doi:10.1016/j.jbc.2022.102239)
Supplement: Supporting figures S1−S6 [file mmc1.pdf]

Supporting Information

Supplementary Figures 1-5.

Supplementary Figure 1:

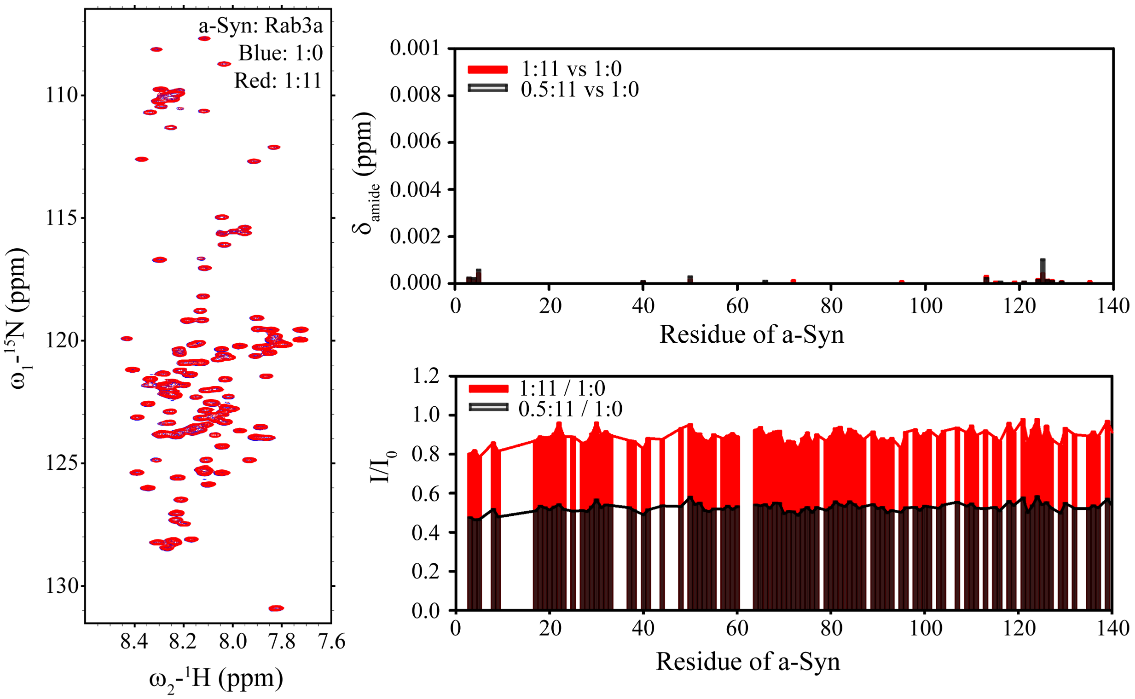

Supplementary Figure 1: Salt eliminates chemical shift changes in the C-terminus changes of a-Syn upon titration of GTP-bound Rab3a. NMR ^{1}H-^{15}N HSQC spectra of ^{15}N-labeled a-Syn in the absence (1:0, blue) or presence of 11-fold excess (1:11, red) GTP-bound Rab3a exhibit negligible change in the presence of 50 mM NaCl (left panel). No chemical shift changes of the amide cross peaks ($\Delta\delta_{amide} = \sqrt{} 1/2(\Delta\delta_{HN}^2 + (\Delta\delta_N/5)^2)$) upon addition of 11-fold (1:11, red) or 22-fold (0.5:11, black) excess Rab3a are observed at the C-terminus of the protein (right upper panel). No changes of NMR signal intensity ratios in the presence of 11-fold (1:11, red) or 22-fold (0.5:11, black) excess Rab3a are observed at the C-terminus (right lower panel).

Supplementary Figure 2:

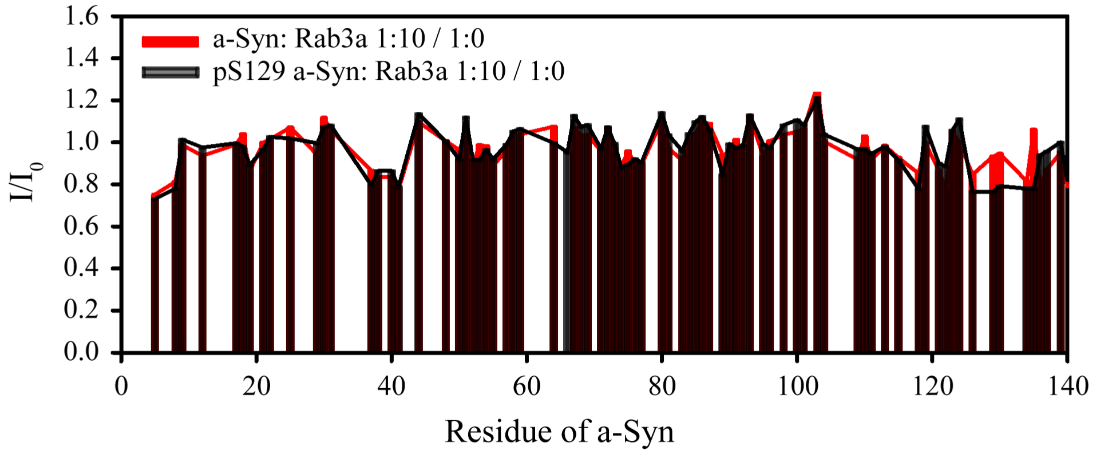

Supplementary Figure 2: Addition of unlabeled Rab3a to ^{15}N-labeled pS129 a-Syn results in a slightly greater decrease in the intensity of C-terminal a-Syn residues (black) than for WT a-Syn (red).

Supplementary Figure 3:

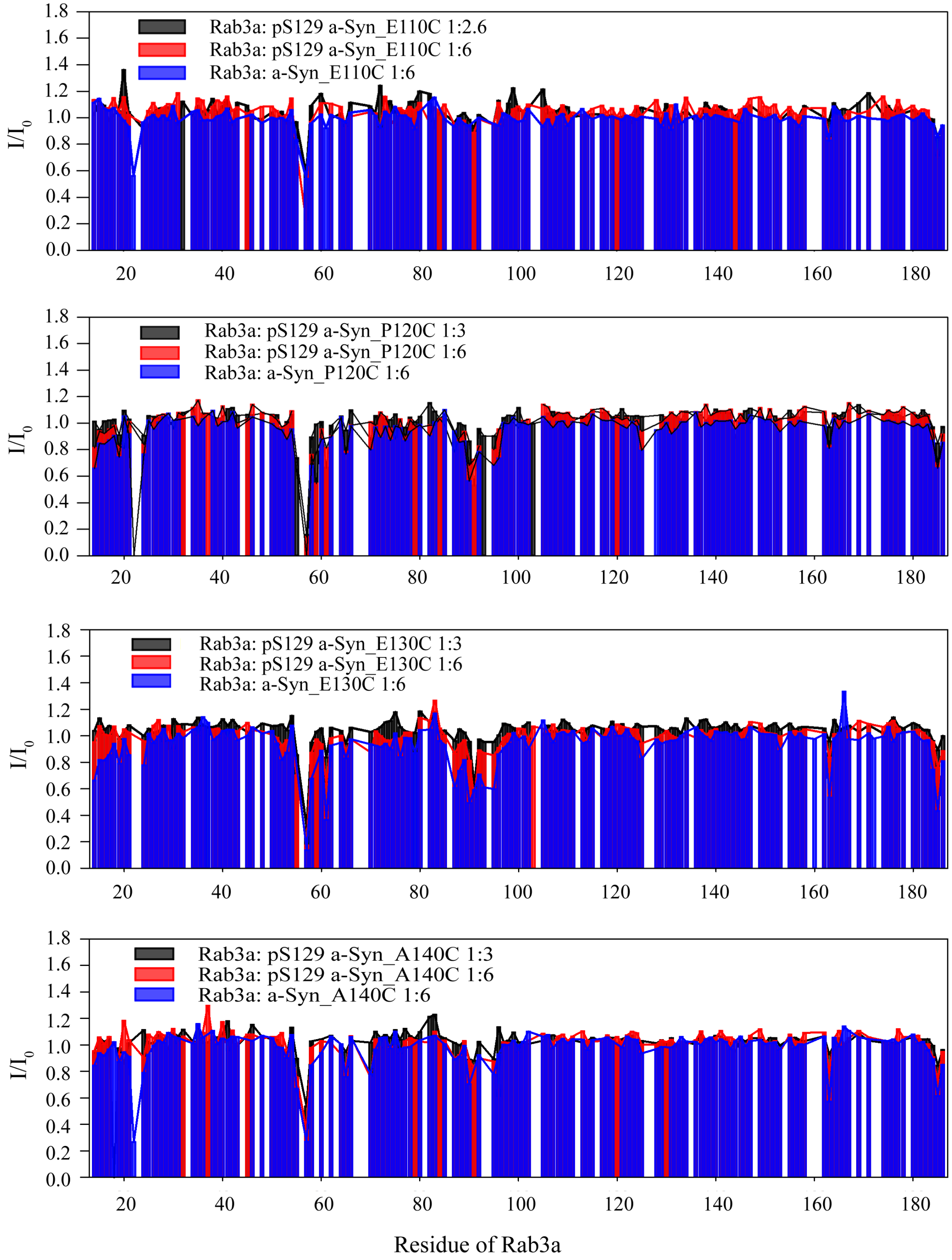

Supplementary Figure 3: Addition of spin-labeled ^{14}N-pS129 a-Syn to ^{15}N-labeled Rab3a results in PREs for Rab3a residues (black, red) similar to those observed for addition of WT a-^{14}N-Syn (blue).

Supplementary Figure 4:

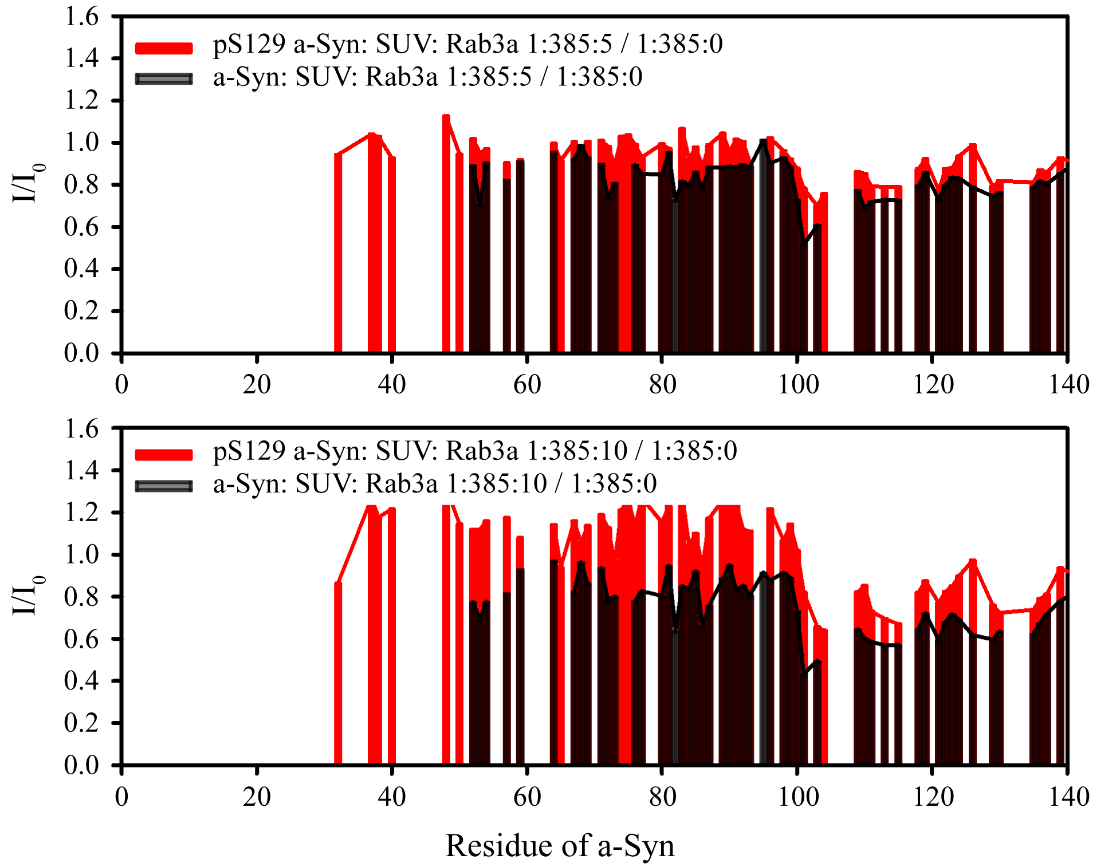

Supplementary Figure 4: In the presence of SUVs and SUV-anchored Rab3a, pS129 a-Syn (red) exhibits slightly decreased membrane binding and Rab3a association, as indicated by higher intensity ratios (with/without Rab3a) in both the N-terminal lipid-binding domain and the C-terminal tail compared with unmodified a-Syn (black) at a-Syn:SUV:Rab3a stoichiometries of 1:385:5 (top) and 1:385:10 (bottom).

Supplementary Figure 5:

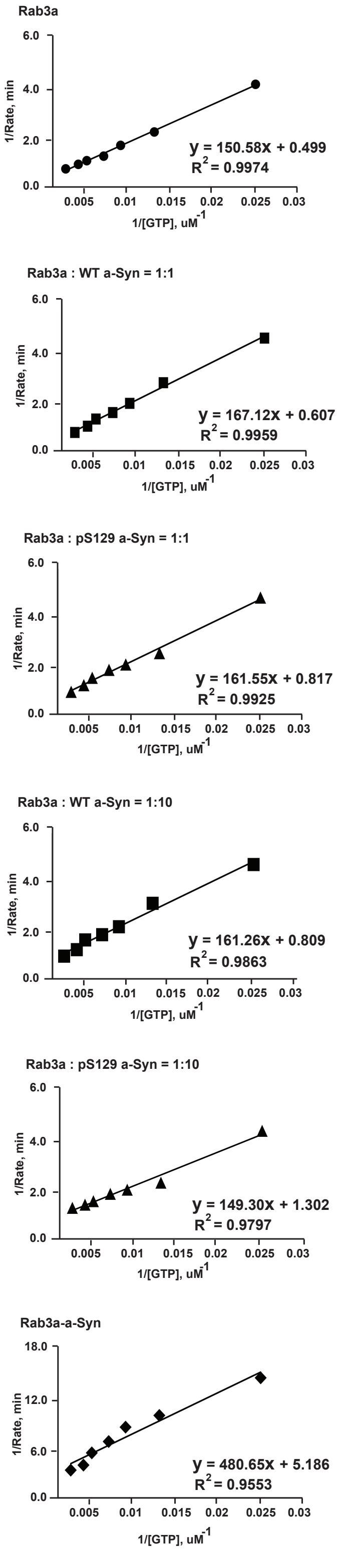

Supplementary Figure 5: Lineweaver Burke plots of Rab3a activity in the absence or presence of 1:1 or 10:1 WT or pS129 a-Syn or for the Rab3a-a-Syn fusion construct.

Supplementary Figure 6:

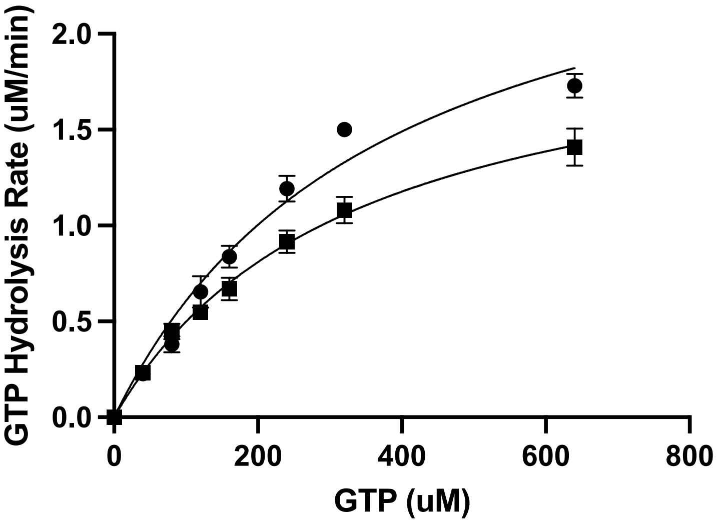

Supplementary Figure 6: Rab3a GTPase activity, measured by phosphate release as a function of time at different GTP concentrations, is decreased when Rab3a is anchored to SUVs via an N-terminal His-tag (squares) vs free in solution (circles). Measurements were performed at 37 °C at a final Rab3a concentration of 0.5 μM. Solid lines represent non-linear least squares fits of the data to the Michaelis Menten equation.
